# *CDK6* 3'UTR polymorphisms alter the susceptibility to cervical cancer among Uyghur females

**DOI:** 10.1002/mgg3.626

**Published:** 2019-03-04

**Authors:** Kailibinuer Aierken, Zhihong Dong, Tangnuer Abulimiti, Yuanyuan Zhang, Guzhalinuer Abuduxikuer, Gulixian Tuerxun, Guligeina Abudurexiti, Aziguli Maimaitiaishan, Patiman Mijiti, Guzhalinuer Abulizi

**Affiliations:** ^1^ 5th Department of Gynecology Affiliated Tumor Hospital of Xinjiang Medical University Urumqi China; ^2^ Outpatient Department Affiliated Tumor Hospital of Xinjiang Medical University Urumqi China

**Keywords:** 3' untranslated region, *CDK6* gene, cervical cancer risk, single nucleotide polymorphisms, Uyghur females

## Abstract

**Aims:**

Cyclin dependent kinase 6 (CDK6) plays a crucial role in malignant tumor whereas less is reported in cervical cancer development. The aim of this study was to evaluate the effects of *CDK6* 3' untranslated region (3'UTR) polymorphisms on cervical cancer susceptibility among Uyghur females.

**Methods:**

The genotypes of the six *CDK6* variants (rs8179, rs42032, rs42033, rs42034, rs42035, and rs42038) were identified among 306 cervical cancer cases and 310 healthy controls with the Agena MassARRAY platform. The associations of the candidate single nucleotide polymorphisms (SNPs) with the cervical cancer risk were evaluated under genetic models using conditional logistic regression analysis. Bioinformatics analysis was performed for SNP function prediction with the online databases. The expression differences between tumor tissues and normal cervix samples were also examined by Real‐time PCR.

**Results:**

*CDK6* rs8179 and rs42033 were correlated to the decreased risk of cervical cancer in Uyghurs under the allele model (rs8179 and rs42033: OR = 0.60, 95% CI: 0.37–0.99, *p* = 0.043) and log‐additive model (rs8179 and rs42033: OR = 0.62, 95% CI: 0.38–1.00, *p* = 0.047). Rs8179, rs42032, and rs42033 were associated with susceptibility to high‐grade cervical cancer in different genetic models as well (*p* < 0.05). Dataset‐based analysis also uncovered the potential effects of these significant SNPs. In addition, aberrant expression of *CDK6* were detected in cervical tumors.

**Conclusions:**

Our results suggested the relationships between *CDK6* 3'UTR polymorphisms and cervical cancer pathogenesis, and the involvement of *CDK6* in cervical cancer development among Uyghur females.

## 1 INTRODUCTION

With a significant estimated incidence (570,000) and mortality (311,000) worldwide, cervical cancer ranks as the fourth most common cancer and the leading cause of cancer death in females (Bray et al., 2018). Although the number of new cases has decreased in numerous populations during the past decades, the incidence of cervical cancer continues to climb in China, especially among younger women in rural (Xueting et al., 2017). The Uyghur, vast majority of whom lives in the Xinjiang Uyghur Autonomous Region of China, has a higher prevalence of cervical cancer than other ethnic groups, as well as the highest mortality rate among minorities (Abulizi et al., 2018). However, the pathogenesis of cervical cancer is incompletely understood. Research on cervical cancer etiology has demonstrated the involvement and necessity of persistent human papillomavirus (HPV) infection and chronic inflammation in cervical malignancy, which are affected by other environmental co‐factors including oral contraceptives, parity and tobacco smoking (Boda et al., 2018; Bosch, Lorincz, Muñoz, Meijer, & Shah, 2002; Castellsagué, Bosch, & Muñoz, 2002). Recently, accumulating evidence has highlighted the causative roles of genetic determinants in cervical tumorigenesis. Single nucleotide polymorphisms (SNPs) based investigation has corroborated the associations of the variants with cervical cancer risk in different populations (Dardiotis et al., 2018; Shi et al., 2014; Wang & Luo, 2018). Previous work performed by Dardiotis et al. have revealed that rs4737999 in *SULF1* gene was associated with the development of precancerous lesions and cervical cancer among females from Greece (Dardiotis et al., 2018). Shi et al. have demonstrated that the CC genotype of IL‐6 −174 and −572 might confer an increased risk of cervical cancer because of the higher frequency in cervical cancer patients compared with healthy controls (Shi et al., 2014). Wang et al. have provided the significant evidence of the influence of LINC00673 rs11655237 polymorphism on cervical cancer risk among Chinese females (Wang & Luo, 2018). These results strongly imply the genetic predisposition for individuals to cervical cancer and yield new insights on its pathogenesis.

Cyclin‐dependent kinases (CDKs) are a family of protein kinases whose roles have been reported in cell division, apoptosis and neurogenesis (Malumbres, 2014; Sherr & Roberts, 2004). Among all the members, CDK1, CDK2, CDK4 and CDK6 are implicated in cell cycle regulation while CDKs7‐11 are relevant to transcription (Malumbres & Barbacid, 2005). The enzymatic activity of CDKs can be stimulated by binding to cyclins and the deregulation of this function is a hallmark of several diseases, including cancer (Tadesse, Yu, Kumarasiri, Le, & Wang, 2015). Previous research has elucidated that the disorder of CDKs may lead to the malignant proliferation of tumor cells and tumorigenesis (Malumbres & Barbacid, 2009; Tadesse et al., 2015). Cyclin‐dependent kinase 6 (CDK6) is a member of CDK family, which has been identified not only as a CDK but a transcriptional regulator (Kollmann et al., 2013; Uras et al., 2016). CDK6 is responsible for G1 to S cell‐cycle regulation and cell differentiation, and the abnormal expression pattern of this gene has been reported in diverse cancers, such as colorectal carcinoma, medulloblastoma, and oral squamous cell carcinomas (Andisheh‐Tadbir, Ashraf, & Jeiroodi, 2018; Tadesse et al., 2015). Specifically, CDK6 has been recognized as a novel transcriptional regulator in acute lymphoid leukemia and acute myeloid leukemia. The aberrant activity of CDK6‐cyclin complex has been detected in hematopoietic malignancies (Scheicher et al., 2015). These results provide the evidence for the crucial role of CDK6 in cancer development and we could hypothesize that there might be some relationships between CDK6 and cervix oncogenesis. Moreover, genetic variations in CDKs have been discussed as potential risk factors conferring susceptibility to cancer whereas the influence of *CDK6* polymorphisms is less reported (Kibel et al., 2016). Research on the susceptible SNPs is an important step towards the understanding of the *CDK6 *in cervical cancer development.

3' Untranslated region (3'UTR) plays a crucial role in translation control, mRNA degradation, and subcellular localization (Mignone, Gissi, Liuni, & Pesole, 2002). The interaction of microRNA “seed sequence” and gene 3'UTR has been well‐studied at post‐transcriptional level (Lee & Vasudevan, 2013). Therefore, it could be reasonably speculated that the SNPs in gene 3'UTR contribute to the aberrant modulation of mRNAs, which have been proved to be implicated in different malignancies (Liu et al., 2013; Wu et al., 2018). In this study, a case‐control study was carried out in order to investigate the potential impact of the polymorphisms in *CDK6* 3'UTR on individual susceptibility to cervical cancer, which is still an obvious public health threat to Uyghur females from Xinjiang province, China (Abulizi et al., 2018). The in‐depth genetic information obtained from the findings could not only enhance our comprehension of the *CDK6* in cervical cancer, but provide new targets for cancer assessment, prevention and prognosis in Uyghur population.

## 2 MATERIALS AND METHODS

### 2.1 Ethics statement

Our study was approved by the ethics committee from the Affiliated Tumor Hospital of Xinjiang Medical University. All procedures were carried out in accordance with the ethical standards of the ethics committee and with the 1964 Helsinki declaration, and its later amendments. Informed consent was signed by each subjects prior to blood and tissue samples collection.

### 2.2 Study population

An initial sample comprised of 306 cases was recruited from the Affiliated Tumor Hospital of Xinjiang Medical University, with histopathological confirmation as cervical cancer patients by at least two pathologists. International Federation of Gynecology and Obstetrics (FIGO) stage and clinical differentiated degree (low‐grade and high‐grade) were also investigated at enrollment for analysis. It was noteworthy that patients with systemic or topical treatments and other diseases were excluded from our research. Additionally, eligible females who matched the age and ethnicity of the cases were consecutively involved from the health examination center of the same hospital, and were regarded as the controls. Finally, 310 unrelated, healthy individuals with no history of gynecologic tumors and diseases were included in this study. All the participants belonged to Uyghur minority from Xinjiang province, northwest China, and had at least three generations of Uyghur ancestry.

### 2.3 RNA extraction and expression examination

Total RNA was extracted from 50 cervical cancer tissues and 50 normal cervix samples by Trizol methods used in our laboratory, and quantified with Nanodrop 2000 UV spectrophotometer (Thermo Scientific, Waltham, MA). Using the PrimeScrip RT Master Mix (Perfect Real Time) (TaKaRa, Kusatsu, Japan), cDNA was synthesized from the template RNA. Real time PCR was performed to determine the expression of *CDK6* gene with the TB Green Premix Ex Taq II (Tli RNaseH Plus) (TaKaRa, Kusatsu, Japan). PCR amplification and fluorescence detection were carried out on ABI 7500 Fast Real‐Time System (Applied Biosystems, CA). The relatively levels of the *CDK6* gene were calculated by 2^‐ΔΔCt^ method with the *GAPDH* as normalization.

### 2.4 Genomic DNA isolation and SNP genotyping

Genomic DNA was extracted from the peripheral blood samples provided by the experimental subjects using the classical phenol‐chloroform method. The concentration and quality of the purified DNA were measured with Nanodrop 2000 UV spectrophotometer (Thermo Scientific, Waltham, MA). After searching the genetic information provided by dbSNP database (https://www.ncbi.nlm.nih.gov/snp/) and 1,000 Genomes database (http://www.internationalgenome.org/), SNPs whose minor allele frequency (MAF) beyond 5% in Asian populations were preliminarily selected in order to achieve adequate statistical power. In genetic association studies, we usually use a small number of tag‐SNPs to represent the genetic variation of the adjacent regions for disease risk evaluation. Moreover, *r* ^2^ = 0.8 was used as the evaluation standard for tag‐SNP selection. Finally, rs8179, rs42032, rs42033, rs42034, rs42035, and rs42038 in the 3'UTR of the *CDK6 *gene were eventually selected as candidate SNPs for further genotype identification and risk association analysis. Agena Bioscience Assay Design Suite software, version 2.0 (https://agenacx.com/online-tools/) was applied for MassARRAY assay design. The SNP genotype was identified by using the MassARRAY Nanodispenser and MassARRAY iPLEX method (Agena Bioscience, San Diego, CA, USA) according to the manufacturer's instructions. The genome regions containing the SNP sites were amplified by multiplex PCR assays. During this process, a single “mass‐modified” terminator nucleotide base that is specifically complementary to the polymorphic site was added to the end of the amplified fragment. Therefore, the expected mass for the fragment was dependent on which polymorphic base was present, and could be further calculated. Subsequently, the multiplex analyte mixture obtained from PCR was transferred to a SpectroCHIP Array using the purpose‐built dispenser Agena Bioscience Nanodispenser RS1000. With the matrix‐assisted laser desorption/ionization—time of flight (MALDI‐TOF) mass spectrometry of the MassARRAY iPLEX platform, the mass of the DNA fragments was differentiated according to the relative time of flight. Finally, the genotyping results at the polymorphic site were managed and outputted by Agena Bioscience TYPER software, version 4.0.

### 2.5 Statistical analysis

Age distribution and *CDK6* expression differences between cervical cancer cases and healthy controls were estimated by the independent sample *t* test. The *p* value >0.05 means the match of age between the case and control groups. The departure from Hardy–Weinberg equilibrium (HWE) was assessed by comparing the observed and expected heterozygosity in controls with Fisher's exact test. HWE *p* value >0.05 indicated that the SNP was eligible for following statistical tests. All the basic statistical analysis was carried out using SPSS 19.0 (SPSS, Chicago, IL, USA) and Microsoft Excel, and *p* < 0.05 was regarded as statistical significance. Furthermore, the risk association study was performed in multiple inheritance models using SNPstats software (http://bioinfo.iconcologia.net/SNPstats; Jiri et al., 2013; Peng et al., 2015). Odds ratio (OR) values and 95% confidence intervals (CIs) were calculated basing on the conditional logistic regression with adjustment for age (Bland & Altman, 2000; Zhou et al., 2015). Linkage disequilibrium (LD) blocks were constructed with Haploview, version 4.2 and the associations of different haplotypes with cervical cancer risk were evaluated by logistic regression model as well (Barrett, Fry, Maller, & Daly, 2005). The LD patterns among the genetic variations were first assessed with the genotyping results. In this process, the SNP pairwise distance>500 kb was ignored. The degree of LD was measured by *r* ^2^ and *D*′ (ranging from 0 to 1). *D*′ = 1 is considered as complete linkage disequilibrium. The specific collection of alleles of variants in a LD block is regarded as haplotype. Haplotypes with frequency greater than 5% were evaluated on their associations with the cervical cancer risk using available OR (95% CI) and *p* value on SHEsis platform.

### 2.6 Bioinformatics analysis

We obtained the functional information and annotation of the valuable variants using the HaploReg v4.1 database (https://pubs.broadinstitute.org/mammals/haploreg/haploreg.php). Owing to the fact that the selected SNPs in this study were resided in the 3'UTR of *CDK6*, SNPinfo Web Server (https://snpinfo.niehs.nih.gov/) was employed to predict the microRNAs whose binding sites contain rs8179, rs42032, and rs42033.

## 3 RESULTS

### 3.1 Characteristics of the study subjects

In the current study, we achieved a well matching of cases and controls for age and sample size (*p* > 0.05). Totally, 306 cervical cancer patients and 310 healthy participants were eventually enrolled with a mean age of 52.75 and 52.42 respectively (Table 1). Moreover, the frequency distribution of the cervical cancer cases regarding to FIGO stage and differential degree was calculated and listed in Table 1.

**Table 1:** Characteristics of the cervical cancer patients and healthy controls in this study

Characteristics	Cervical cancer cases (*N* = 306)	Healthy controls (*N* = 310)	*p* Value
Age (years, mean ± *SD*)	52.75 ± 11.41	52.42 ± 12.12	0.724*
FIGO stage (%)			
I	44 (14.4%)		
II	158 (51.6%)		
III	86 (28.1%)		
IV	5 (1.6%)		
Absence	13 (4.2%)		
Differentiated degree (%)			
Low‐grade	79 (25.8%)		
High‐grade	135 (44.1%)		
Non‐keratinized	3 (1.0%)		
Absence	89 (29.1%)		

Note

*SD*: Standard deviation; FIGO: International Federation of Gynecology and Obstetrics.

* *p* Value was calculated with independent samples *t* test.

### 3.2 *CDK6* expression level in cervical cancer

**Figure 1:**
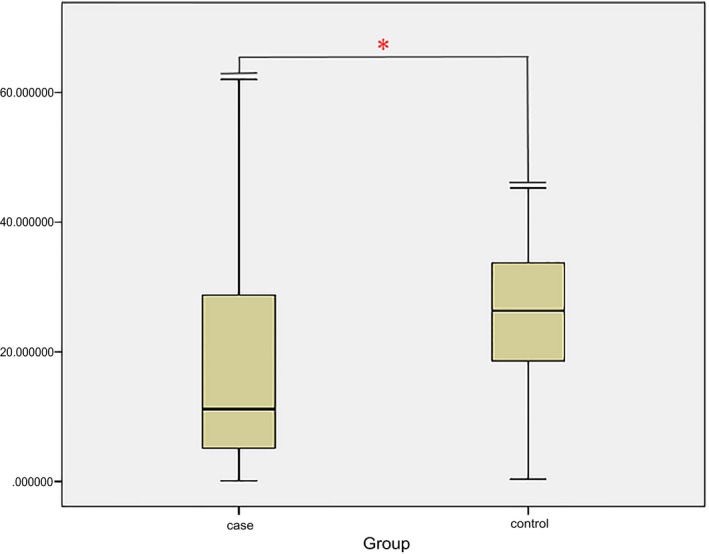
*CDK6* expression levels in 50 Uyghur cervical cancer tissues and 50 normal cervix samples. The expression pattern of *CDK6* gene was detected by real time PCR in 50 cervical tumors and 50 cervix samples. The level of *CDK6* was significantly down‐regulated in cervical tumors when compared with controls (*p* < 0.05). Asterisk indicates the significant difference

The mRNA levels of *CDK6* were examined in cervical cancer tissues from Uyghur patients and normal cervix samples. Interestingly, significant down‐regulation of *CDK6* appeared in cervical cancer cases, which suggested the suppression effects of *CDK6* during tumor development (Figure 1).

### 3.3 Basic information for the candidate SNPs

Descriptive information, including chromosome, position, allele, role, MAF and HWE *p* value were presented for each selected SNP (rs8179, rs42032, rs42033, rs42034, rs42035, and rs42038) in Table 2. All the variants were located in the *CDK6 *3'UTR with MAF ranged from approximately 0.013 to 0.115. HWE *p* values were also obtained with exact test, which suggested the inclusion of rs8179, rs42032, rs42033, rs42034, and rs42035 in further statistical study (*p* > 0.05) and the exclusion of rs42038 owing to its departure from HWE (*p* < 0.001).

**Table 2:** Basic information of the candidate SNPs in *CDK6*

SNP	Chromosome	Position	Alleles A < B	Role	Minor allele frequency		HWE *p‐*Value
					Case	Control	
rs8179	Chr 7	92,606,850	T < C	3'UTR Variant	0.044	0.071	0.055
rs42032	Chr 7	92,608,112	A < G	3'UTR Variant	0.078	0.105	0.759
rs42033	Chr 7	92,608,219	T < A	3'UTR Variant	0.044	0.071	0.055
rs42034	Chr 7	92,609,830	G < A	3'UTR Variant	0.110	0.115	0.263
rs42035	Chr 7	92,610,217	G < A	3'UTR Variant	0.111	0.115	0.263
rs42038	Chr 7	92,614,405	T < C	3'UTR Variant	0.013	0.023	<0.001*

Note

SNP: Single nucleotide polymorphism; HWE: Hardy‐Weinberg equilibrium; UTR: Untranslated region.

HWE *p*‐value: *p*‐value obtained from Fisher's exact test.

* *p* < 0.05 indicates the deviation from HWE.

### 3.4 *CDK6* polymorphisms and cervical cancer risk in Uyghur females

The relationships between the *CDK6* variations and cervical cancer risk were evaluated in a Uyghur representative group. The allele and genotype frequencies of all SNPs in both case and control groups were summarized in Table S1. According to the statistical results, rs8179 and rs42033, rs42034 and rs42035 shared similar allele and genotype frequencies in our study population. In further association analysis, the allele with lower frequency was hypothesized as a risk factor. As showed in Table 3, significant associations were detected between *CDK6* rs8179 and rs42033, and decreased risk of cervical cancer among Uyghur females in allele model and log‐additive model (allele model: OR = 0.60, 95% CI: 0.37–0.99, *p* = 0.043; log‐additive model: OR = 0.62, 95% CI: 0.38–1.00, *p* = 0.047). The minor allele “T” at rs8179 and rs42033 positions reduced the susceptibility to cervical cancer by about 40% when compared with the wild allele (Table 3). However, there were no associations observed between rs42032, rs42034, rs42035 and cervical cancer risk in this study (Table S2).

**Table 3:** The association of rs8179 and rs42033 with cervical cancer susceptibility in Uygur population

Model	Allele/genotype	Case	Control	OR (95% CI)	*p* Value
rs8179 (*N* = 616, call rate 100%)					
Allele	C	585 (95.6%)	576 (92.9%)	1.00	***0.043***
	T	27 (4.4%)	44 (7.1%)	***0.60 (0.37−0.99)***	
Codominant	C/C	279 (91.2%)	270 (87.1%)	1.00	0.031
	C/T	27 (8.8%)	36 (11.6%)	0.72 (0.43−1.22)	
	T/T	0 (0.0%)	4 (1.3%)	0.00 (0.00–NA)	
Dominant	C/C	279 (91.2%)	270 (87.1%)	1.00	0.100
	C/T‐T/T	27 (8.8%)	40 (12.9%)	0.65 (0.39−1.09)	
Recessive	C/C‐C/T	306 (100.0%)	306 (98.7%)	1.00	0.019
	T/T	0 (0.0%)	4 (1.3%)	0.00 (0.00–NA)	
Log‐additive	—	—	—	***0.62 (0.38−1.00)***	***0.047***
rs42033 (*N* = 616, call rate 100%)					
Allele	A	585 (95.6%)	576 (92.9%)	1.00	***0.043***
	T	27 (4.4%)	44 (7.1%)	***0.60 (0.37−0.99)***	
Codominant	A/A	279 (91.2%)	270 (87.1%)	1.00	0.031
	A/T	27 (8.8%)	36 (11.6%)	0.72 (0.43−1.22)	
	T/T	0 (0.0%)	4 (1.3%)	0.00 (0.00–NA)	
Dominant	A/A	279 (91.2%)	270 (87.1%)	1.00	0.100
	A/T‐T/T	27 (8.8%)	40 (12.9%)	0.65 (0.39−1.09)	
Recessive	A/A‐A/T	306 (100.0%)	306 (98.7%)	1.00	0.019
	T/T	0 (0.0%)	4 (1.3%)	0.00 (0.00–NA)	
Log‐additive	—	—	—	***0.62 (0.38−1.00)***	***0.047***

Note

SNP: Single nucleotide polymorphism; OR: Odds ratio; 95% CI: 95% confidence interval.

Bold italics indicates the SNP with statistical significance (*p* < 0.05).

Upon stratification by tumor grade, we performed analysis with an attempt to ascertain the effects of these variants on tumor differentiation and the results were highlighted in Table 4. In accordance with our statistically significant findings of the allele model, the minor allele of rs8179, rs42032, and rs42033 exerted protective roles in high‐grade cervical cancer development among Uyghur females (rs8179: OR = 0.35, 95% CI: 0.15–0.78, *p* = 0.008; rs42032: OR = 0.43, 95% CI: 0.23–0.80, *p* = 0.006; rs42033: OR = 0.35, 95% CI: 0.15–0.78, *p* = 0.008). Individuals carrying the heterozygous genotype “C/T” in rs8179 (OR = 0.40, 95% CI: 0.17–0.92, *p* = 0.017), “G/A” in rs42032 (OR = 0.47, 95% CI: 0.24–0.88, *p* = 0.012), and “A/T” in rs42033 (OR = 0.40, 95% CI: 0.17–0.92, *p* = 0.017) were less likely to suffer from high‐grade cervical cancer when compared with those possessing the wild homozygous “C/C”, “G/G”, and “A/A” at each locus respectively. The homozygous genotype of minor allele can not be identified in high‐grade cervical cancer cases. Moreover, dominant model and log‐additive model revealed the remarkable relationships of the same SNPs and decreased risk of high‐grade cervical cancer as well (rs8179, dominant model: OR = 0.36, 95% CI: 0.16–0.83, *p* = 0.009; log‐additive model: OR = 0.37, 95% CI: 0.16–0.82, *p* = 0.006; rs42032 dominant model: OR = 0.44, 95% CI: 0.23–0.83, *p* = 0.007; log‐additive model: OR = 0.44, 95% CI: 0.23–0.81, *p* = 0.005; rs42033, dominant model: OR = 0.36, 95% CI: 0.16–0.83, *p* = 0.009; log‐additive model: OR = 0.37, 95% CI: 0.16–0.82, *p* = 0.006). Nevertheless, no evidence was found for the risk associations of rs42034, rs42035 with high‐grade cervical cancer and of all SNPs with low‐grade cervical cancer risk in this study (Table 4 and Table S3).

**Table 4:** Significant associations of rs8179, rs42032 and rs42033 with cervical cancer risk after stratifying by tumor grade

SNP	Model	Allele/Genotype	Low‐grade (*N* = 79) versus controls (*N* = 310)				High‐grade (*N* = 135) versus controls (*N* = 310)			
			Case	Control	OR (95% CI)	*p* Value	Case	Control	OR (95% CI)	*p* Value
rs8179	Allele	C	146 (92.4%)	576 (92.9%)	1.00	0.829	263 (97.4%)	576 (92.9%)	1.00	***0.008***
		T	12 (7.6%)	44 (7.1%)	1.08 (0.55−2.09)		7 (2.6%)	44 (7.1%)	***0.35 (0.15−0.78)***	
	Codominant	C/C	67 (84.8%)	270 (87.1%)	1.00	0.300	128 (94.8%)	270 (87.1%)	1.00	***0.017***
		C/T	12 (15.2%)	36 (11.6%)	1.34 (0.66−2.72)		7 (5.2%)	36 (11.6%)	***0.40 (0.17−0.92)***	
		T/T	0 (0.0%)	4 (1.3%)	0.00 (0.00–NA)		0 (0.0%)	4 (1.3%)	0.00 (0.00–NA)	
	Dominant	C/C	67 (84.8%)	270 (87.1%)	1.00	0.610	128 (94.8%)	270 (87.1%)	1.00	***0.009***
		C/T‐T/T	12 (15.2%)	40 (12.9%)	1.20 (0.60−2.42)		7 (5.2%)	40 (12.9%)	***0.36 (0.16−0.83)***	
	Recessive	C/C‐C/T	79 (100.0%)	306 (98.7%)	1.00	0.180	135 (100.0%)	306 (98.7%)	1.00	0.100
		T/T	0 (0.0%)	4 (1.3%)	0.00 (0.00–NA)		0 (0.0%)	4 (1.3%)	0.00 (0.00–NA)	
	Log‐additive	—	—	—	1.07 (0.56−2.02)	0.840	—	—	***0.37 (0.16−0.82)***	***0.006***
rs42032	Allele	G	136 (86.1%)	555 (89.5%)	1.00	0.221	257 (95.2%)	555 (89.5%)	1.00	***0.006***
		A	22 (13.9%)	65 (10.5%)	1.38 (0.82−2.32)		13 (4.8%)	65 (10.5%)	***0.43 (0.23−0.80)***	
	Codominant	G/G	58 (73.4%)	249 (80.3%)	1.00	0.410	122 (90.4%)	249 (80.3%)	1.00	***0.012***
		G/A	20 (25.3%)	57 (18.4%)	1.50 (0.84−2.70)		13 (9.6%)	57 (18.4%)	***0.47 (0.24−0.88)***	
		A/A	1 (1.3%)	4 (1.3%)	1.09 (0.12−9.94)		0 (0.0%)	4 (1.3%)	0.00 (0.00–NA)	
	Dominant	G/G	58 (73.4%)	249 (80.3%)	1.00	0.190	122 (90.4%)	249 (80.3%)	1.00	***0.007***
		G/A‐A/A	21 (26.6%)	61 (19.7%)	1.48 (0.83−2.62)		13 (9.6%)	61 (19.7%)	***0.44 (0.23−0.83)***	
	Recessive	G/G‐G/A	78 (98.7%)	306 (98.7%)	1.00	1.000	135 (100.0%)	306 (98.7%)	1.00	0.100
		A/A	1 (1.3%)	4 (1.3%)	1.00 (0.11−9.08)		0 (0.0%)	4 (1.3%)	0.00 (0.00–NA)	
	Log‐additive	—	—	—	1.38 (0.82−2.32)	0.230	—	—	***0.44 (0.23−0.81)***	***0.005***
rs42033	Allele	A	146 (92.4%)	576 (92.9%)	1.00	0.829	263 (97.4%)	576 (92.9%)	1.00	***0.008***
		T	12 (7.6%)	44 (7.1%)	1.08 (0.55−2.09)		7 (2.6%)	44 (7.1%)	***0.35 (0.15−0.78)***	
	Codominant	A/A	67 (84.8%)	270 (87.1%)	1.00	0.300	128 (94.8%)	270 (87.1%)	1.00	***0.017***
		A/T	12 (15.2%)	36 (11.6%)	1.34 (0.66−2.72)		7 (5.2%)	36 (11.6%)	***0.40 (0.17−0.92)***	
		T/T	0 (0.0%)	4 (1.3%)	0.00 (0.00–NA)		0 (0.0%)	4 (1.3%)	0.00 (0.00–NA)	
	Dominant	A/A	67 (84.8%)	270 (87.1%)	1.00	0.610	128 (94.8%)	270 (87.1%)	1.00	***0.009***
		A/T‐T/T	12 (15.2%)	40 (12.9%)	1.20 (0.60−2.42)		7 (5.2%)	40 (12.9%)	***0.36 (0.16−0.83)***	
	Recessive	A/A‐A/T	79 (100.0%)	306 (98.7%)	1.00	0.180	135 (100.0%)	306 (98.7%)	1.00	0.100
		T/T	0 (0.0%)	4 (1.3%)	0.00 (0.00–NA)		0 (0.0%)	4 (1.3%)	0.00 (0.00–NA)	
	Log‐additive	—	—	—	1.07 (0.56−2.02)	0.840	—	—	***0.37 (0.16−0.82)***	***0.006***

Note

SNP: Single nucleotide polymorphism; OR: Odds ratio; 95% CI: 95% confidence interval.

Bold italics indicates the SNP with statistical significance (*p* < 0.05).

**Figure 2:**
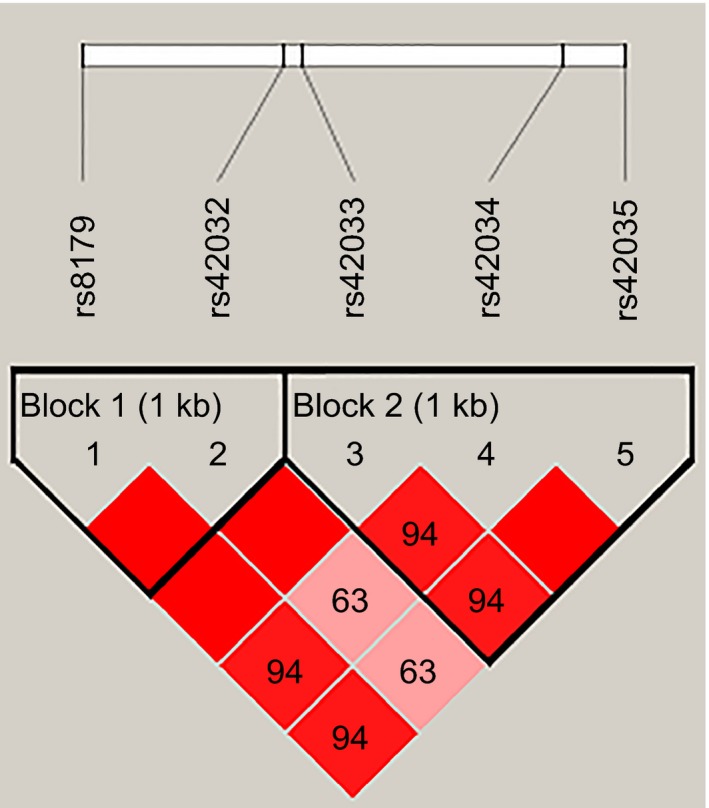
Linkage disequilibrium block construction. Two blocks were detected in *CDK6* gene. Block 1: rs8179‐rs42032; Block 2: rs42033‐rs42034‐rs42035. The LD degree is displayed by standard color schemes with bright red for very strong LD and pink red for relatively weaker LD

Furthermore, haplotype analyses were performed and two dependent LD blocks were found in *CDK6* gene, formed by rs8179‐rs42032, and rs42033‐rs42034‐rs42035 respectively. In Figure 2, the LD pattern is showed by standard color schemes. The bright red indicates very strong LD. Haplotypes with frequency more than 0.05 were involved in the subsequent analysis whereas the results did not yield any statistical evidence of the associations between them and cervical cancer risk in Uyghur population (*p* > 0.05; Table 5).

**Table 5:** Haplotype analysis of the *CDK6* variants

Chromosome	Gene	Block	Haplotype	OR (95% CI)	*p* Value
chr7	CDK6	rs8179|rs42032	TA	0.62 (0.38−1.00)	0.051
			CA	1.02 (0.54−1.91)	0.953
			CG	0.73 (0.49−1.08)	0.110
chr7	CDK6	rs42033|rs42034|rs42035	TGG	0.65 (0.40−1.06)	0.082
			AGG	1.52 (0.91−2.52)	0.107
			AAA	0.94 (0.66−1.33)	0.729

Note

SNP: Single nucleotide polymorphism; OR: Odds ratio; 95% CI: 95% confidence interval.

### 3.5 SNP functional annotation

In order to explore the potential roles of the promising SNPs, we conducted functional analysis with HaploReg 4.1 database. As summarized in Table S4, rs8179, rs42032, and rs42033 in *CDK6* were predicted with diverse functions. SNPinfo database also provided us several putative miRNAs that were able to target the 3'UTR sequences containing the three genetic variants (Table S4).

## 4 DISCUSSION

This study first demonstrated the relationships between the 3'UTR variants in *CDK6* gene and cervical cancer susceptibility in Uyghur females from Xinjiang Uyghur Autonomous Region of China. We found that rs8179, rs42032, and rs42033 were significantly associated with the cervical cancer risk among Uyghur females. With bioinformatics prediction, all the three SNPs harbored various functions, and might influence the complementary targeting of putative miRNAs. Additionally, our results also revealed an abnormal expression of *CDK6* in cervical cancer patients with Uyghur descent. These results suggested the significant role of *CDK6* in cervical cancer development in Uyghur.

CDK6 is a vital factor in mediating G_1_/S transition in cell cycle, and is linked to the tumor progression as well (Choi & Anders, 2014; Costello et al., 1997). The adverse impacts of CDK6 have been investigated in most gynecologic tumors, including epithelial ovarian cancer and breast cancer, and its activity is always considered as an important point for tumor inhibition during clinical treatment (Dai et al., 2015; Dall'Acqua et al., 2017; Wolff, 2016). Moreover, research focusing on genetic epidemiology has uncovered that the host variations in *CDK6* contribute to different clinical outcomes among breast cancer patients, which highlights the importance of the study on genetic susceptibility (Dai et al., 2015). The candidate SNPs, namely rs8179, rs42032, and rs42033, were pathogenic factors in our study and found to modulate the risk to high‐grade cervical cancer in Uyghur population after stratified analysis. Carriers with the minor allele at these sites have lower predisposition to cervical cancer. These SNPs indicated the outstanding importance of *CDK6* in high‐grade cervical cancer, and could be employed as clinical predictors for developing the high‐grade tumor among Uyghur females.

3'UTR has been acknowledged to be involved in the gene expression regulation at post‐transcriptional level (Mignone et al., 2002). As a region containing multiple functional sequence elements, 3'UTR are known to modulate the translation, degradation and subcellular localization of the mRNAs via interacting with RNA‐binding proteins or non‐coding RNAs (Jansen, 2001; Mignone et al., 2002). The mutations in 3'UTR have been detected and reported to confer serious pathology (Conne, Stutz, & Vassalli, 2000). Remarkably, rs8179, rs42032, and rs42033 are mapped to the 3'UTR of *CDK6*. Considering the regulatory roles of 3'UTR and multiple predicted functions of the promising SNPs in this work, we speculated that the significant variants rs8179, rs42032, and rs42033 were involved in the mediation of the 3' end of *CDK6* mRNA, by which the variations may disturb the normal regulation of the functional region and lead to the different susceptibility to cervical cancer development. Furthermore, as the fact that these variations are predicted to be resided in the targeting sequence of several miRNAs, the single nucleotide substitution at rs8179, rs42032, and rs42033 are presumed to disturb the interaction of 3'UTR with these specific miRNAs. This process may change the translation efficiency of the *CDK6 *mRNA and alter the subsequent reactions that facilitate the development of malignant tumor. Therefore, our findings directed the next step for the mechanism study of *CDK6* polymorphisms in cervical cancer.

Several limitations still existed in this study. First, the down‐regulated expression of *CDK6* was detected in this work, and the influence of the SNPs on *CDK6* gene expression need to be further explored. Second, the putative function and underlying mechanism of the polymorphisms on *CDK6* mRNA regulation was not clarified. Third, other clinical and exposure information was lacking. Accordingly, further well‐designed studies should be considered to improve the understanding of the roles of *CDK6* and its polymorphisms in cervical cancer among Uyghur females.

Despite the limitations, our research first validated the involvement of *CDK6* in the pathogenesis of cervical cancer, and discovered the associations of *CDK6* variants with cancer risk in Uyghur population. These single polymorphic markers are supposed to serve as new targets for cervical cancer early assessment and prevention among Uyghur females in future.

## CONFLICT OF INTEREST

The authors declare no competing financial interests.
