# Supplementary material for: CDK6 3'UTR polymorphisms alter the susceptibility to cervical cancer among Uyghur females
Source: Mol Genet Genomic Med. 2019 Mar 4;7(5):e626. doi: 10.1002/mgg3.626 (PMC6503018; doi:10.1002/mgg3.626)
Supplement: Supplementary file 3 [file MGG3-7-e626-s003.docx]

Supplementary Table S3 Stratified analyses of rs42034 and rs42035 by tumor grade

| SNP | Model | Allele/Genotype | Low-grade (N = 79) vs. controls (N = 310) | | | | High-grade (N = 135) vs. controls (N = 310) | | | |
| --- | --- | --- | --- | --- | --- | --- | --- | --- | --- | --- |
|  |  |  | Case | Control | OR (95% CI) | *p* value | Case | Control | OR (95% CI) | *p* value |
| rs42034 | Allele | A | 137 (86.7%) | 549 (88.5%) | 1.00 | 0.523 | 247 (91.5%) | 549 (88.5%) | 1.00 | 0.191 |
|  |  | G | 21 (13.3%) | 71 (11.5%) | 1.19 (0.70-2.00) |  | 23 (8.5%) | 71 (11.5%) | 0.72 (0.44-1.18) |  |
|  | Codominant | A/A | 58 (73.4%) | 245 (79.0%) | 1.00 | 0.100 | 112 (83.0%) | 245 (79.0%) | 1.00 | 0.120 |
|  |  | A/G | 21 (26.6%) | 59 (19.0%) | 1.50 (0.84-2.67) |  | 23 (17.0%) | 59 (19.0%) | 0.86 (0.50-1.46) |  |
|  |  | G/G | 0 (0.0%) | 6 (1.9%) | 0.00 (0.00-NA) |  | 0 (0.0%) | 6 (1.9%) | 0.00 (0.00-NA) |  |
|  | Dominant | A/A | 58 (73.4%) | 245 (79.0%) | 1.00 | 0.290 | 112 (83.0%) | 245 (79.0%) | 1.00 | 0.360 |
|  |  | A/G-G/G | 21 (26.6%) | 65 (21.0%) | 1.36 (0.77-2.41) |  | 23 (17.0%) | 65 (21.0%) | 0.78 (0.46-1.33) |  |
|  | Recessive | A/A-A/G | 79 (100.0%) | 304 (98.1%) | 1.00 | 0.099 | 135 (100.0%) | 304 (98.1%) | 1.00 | 0.047 |
|  |  | G/G | 0 (0.0%) | 6 (1.9%) | 0.00 (0.00-NA) |  | 0 (0.0%) | 6 (1.9%) | 0.00 (0.00-NA) |  |
|  | Log-additive | --- | --- | --- | 1.18 (0.70-1.98) | 0.530 | --- | --- | 0.74 (0.45-1.21) | 0.210 |
| rs42035 | Allele | A | 137 (86.7%) | 549 (88.5%) | 1.00 | 0.523 | 247 (91.5%) | 549 (88.5%) | 1.00 | 0.191 |
|  |  | G | 21 (13.3%) | 71 (11.5%) | 1.19 (0.70-2.00) |  | 23 (8.5%) | 71 (11.5%) | 0.72 (0.44-1.18) |  |
|  | Codominant | A/A | 58 (73.4%) | 245 (79.0%) | 1.00 | 0.100 | 112 (83.0%) | 245 (79.0%) | 1.00 | 0.120 |
|  |  | A/G | 21 (26.6%) | 59 (19.0%) | 1.50 (0.84-2.67) |  | 23 (17.0%) | 59 (19.0%) | 0.86 (0.50-1.46) |  |
|  |  | G/G | 0 (0.0%) | 6 (1.9%) | 0.00 (0.00-NA) |  | 0 (0.0%) | 6 (1.9%) | 0.00 (0.00-NA) |  |
|  | Dominant | A/A | 58 (73.4%) | 245 (79.0%) | 1.00 | 0.290 | 112 (83.0%) | 245 (79.0%) | 1.00 | 0.360 |
|  |  | A/G-G/G | 21 (26.6%) | 65 (21.0%) | 1.36 (0.77-2.41) |  | 23 (17.0%) | 65 (21.0%) | 0.78 (0.46-1.33) |  |
|  | Recessive | A/A-A/G | 79 (100.0%) | 304 (98.1%) | 1.00 | 0.099 | 135 (100.0%) | 304 (98.1%) | 1.00 | 0.047 |
|  |  | G/G | 0 (0.0%) | 6 (1.9%) | 0.00 (0.00-NA) |  | 0 (0.0%) | 6 (1.9%) | 0.00 (0.00-NA) |  |
|  | Log-additive | --- | --- | --- | 1.18 (0.70-1.98) | 0.530 | --- | --- | 0.74 (0.45-1.21) | 0.210 |

**SNP: Single nucleotide polymorphism; OR: Odds ratio; 95% CI: 95% confidence interval.**

Supplementary Table S4 Functional analysis and miRNA prediction for the selected *CDK6* SNPs based on HaploReg 4.1

| SNP | Chr: Position | HaploReg 4.1 | SNPinfo |
| --- | --- | --- | --- |
| rs8179 | 7: 92606850 | Enhancer histone marks, Motifs Changed Selected eQTL hits | hsa-miR-25, hsa-miR-507, hsa-miR-557, hsa-miR-576-5p, hsa-miR-92b |
| rs42032 | 7: 92608112 | Promoter histone marks, Enhancer histone marks, Motifs changed, Selected eQTL hits | hsa-miR-1290, hsa-miR-191 |
| rs42033 | 7: 92608219 | Promoter histone marks, Enhancer histone marks, Selected eQTL hits | hsa-miR-455-5p, hsa-miR-548b-3p |

**SNP: Single nucleotide polymorphism; eQTL: Expression quantitative trait loci; Chr: chromosome.**
